# Transcriptomic Analysis Pipeline (TAP) for quality control and functional assessment of transcriptomes

**DOI:** 10.21203/rs.3.rs-3390128/v1

**Published:** 2023-10-11

**Authors:** Joseph Boyd, Emily A.W. Nadeau, Sophie Kogut, Princess Rodriguez, Daniel Munteneau, Thomas O’Leary, Sara Filler, Brent Lockwood, Sara Helms Cahan, Seth Frietze

**Affiliations:** University of Vermont; University of Vermont; University of Washington; University of Vermont; University of Vermont; University of Vermont; Providence College; University of Vermont; University of Vermont; University of Vermont

**Keywords:** thermal stress, transcriptome, RNA-seq, polyA + selection, rRNA-depletion, long non-coding RNA, splicing

## Abstract

**Background:**

RNA-sequencing (RNA-seq) has revolutionized the exploration of biological mechanisms, shedding light on the roles of non-coding RNAs, including long non-coding RNAs (lncRNAs), across various biological processes, including stress responses. Despite these advancements, there remains a gap in our understanding of the implications of different RNA-seq library protocols on comprehensive lncRNA expression analysis, particularly in non-mammalian organisms.

**Results:**

In this study, we sought to bridge this knowledge gap by investigating lncRNA expression patterns in *Drosophila melanogaster* under thermal stress conditions. To achieve this, we conducted a comparative analysis of two RNA-seq library protocols: polyA + RNA capture and rRNA-depletion. Our approach involved the development and application of a Transcriptome Analysis Pipeline (TAP) designed to systematically assess both the technical and functional dimensions of RNA-seq, facilitating a robust comparison of these library protocols. Our findings underscore the efficacy of the polyA + protocol in capturing the majority of expressed lncRNAs within the *Drosophila melanogaster* transcriptome. In contrast, rRNA-depletion exhibited limited advantages in the context of *D. melanogaster* studies. Notably, the polyA + protocol demonstrated superior performance in terms of usable read yield and the accurate detection of splice junctions.

**Conclusions:**

Our study introduces a versatile transcriptomic analysis pipeline, TAP, designed to uniformly process RNA-seq data from any organism with a reference genome. It also highlights the significance of selecting an appropriate RNA-seq library protocol tailored to the specific research context.

**Background:**

Advances in next generation sequencing (NGS) technologies enable the comprehensive analysis of genetic sequences of organisms in a relatively cost-effective manner [1, 2]. Among these technologies, RNA-sequencing (RNA-seq) has emerged as a preeminent method to study fundamental biological mechanisms at the level of cells, tissues, and whole organisms. RNA-seq enables the detection and quantification of various RNA populations, including messenger RNA (mRNA) and various species of non-coding RNA, such as long non-coding RNA (lncRNA), as well as an assessment of features including splice junctions in RNA.

A critical step in the generation of RNA-seq libraries is the removal of ribosomal RNA (rRNA), which accounts for up to 80–90% of total RNA [3]. A universal approach and the most widely used protocol involve the selection of polyadenylated (polyA+) RNA to enrich mRNA. Alternatively, rRNA-depletion (rRNA−) may be used to reduce the levels of rRNA in a sample and thereby enhance the detection of both polyadenylated and non-polyadenylated RNAs, such as histone mRNAs, circular RNAs, and various non-coding RNA species [4-6]. Unlike polyA + enrichment, rRNA depletion probes are specific to the study organism; commercial kits are available to deplete rRNA in samples from common mammalian species such as human, mice, and rat and for insects such as *Drosophila melanogaster*, and methods are available that permit custom rRNA-depletion of rRNA from different organisms [7]. Thus, the choice of library preparation protocol can involve significant trade-offs, depending on the biological system being studied. In mammalian systems, rRNA-depletion RNA-seq library protocols have been demonstrated to provide superior detection of non-exonic RNA species including lncRNAs [8, 9]. However, the advantage of performing rRNA-depletion versus polyA+-selection in the detection of lncRNAs by RNA-seq in *D. melanogaster* is not well characterized.

In this study, we investigated the impact of library preparation protocol on discovery, quantification, and detection of changes in both coding and non-coding RNA in *D. melanogaster* under conditions of thermal stress. The cellular stress response is highly conserved, comprising various pathways and proteins that mediate molecular responses to both endogenous and exogenous stressors. These pathways include genes encoding molecular chaperones such as heat shock protein family members (i.e., *Hsp70*), components of the ubiquitin-dependent ER-associated degradation (ERAD) and unfolded protein response (UPR) pathways (i.e., *Fbox6*) [10-12]. Recently, lncRNAs including HOX transcription antisense RNA (HOTAIR) and prostate cancer antigen 3 (PCA3) in human cells have been shown to be upregulated with cellular stress and may play roles to permit cells to cope with stress [13, 14]. Prior studies in *D. melanogaster* have shown that thermal stress can affect specific physiological, biochemical, and cellular processes [15], as well as the expression of certain stress response genes [16-18]. However, our understanding of the underlying regulation of stress responses and their role in cellular and organismal acclimation and developmental responses remain limited by distinct experimental conditions (intensity or time of stress), tissue sampling, and variation across studies in library preparation and downstream bioinformatic analyses.

To generate a standardized methodology for comparison, we developed a Transcriptome Analysis Pipeline (TAP) that enables the comprehensive analysis of technical and functional measures of RNA-seq data, including quality control, differential gene expression, and alternative splicing, using any transcriptome reference index. We used the pipeline to analyze and compare RNA-seq data generated from polyA + and rRNA− libraries derived from adult *D. melanogaster* exposed to increasingly severe cold and heat stress, with the goal of elucidating the relative strengths and weaknesses of the two methods for 1) assessing the extent of transcriptomic response to thermal stress, 2) identifying functional pathways involved in the thermal stress response, 3) detection and differential expression of lncRNAs, and 4) detection of alternative splicing associated with temperature.

## Results

### Transcriptome Analysis Pipeline (TAP) for comprehensive RNA-seq preprocessing

**Figure 1:**
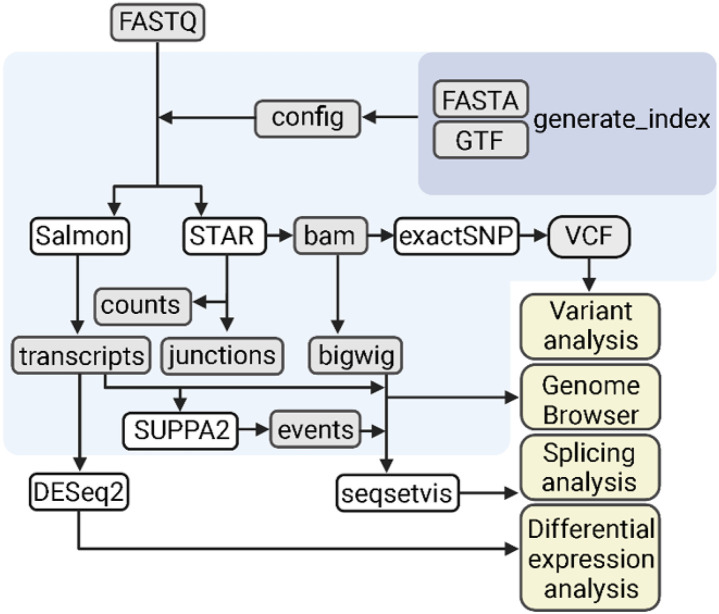
Flowchart representing the Transcriptome Analysis Pipeline (TAP) for processing RNA-seq data. The primary processing steps of TAP are shaded in light blue and the input and output files are shown in grey boxes. The pipeline begins with a generate_index step (shaded in dark blue) that utilizes available reference sequence (FASTA) and gene annotation files (GTF) files to create the required index files for the distinctive processing steps of the pipeline (shown in white boxes). A configuration.csv file lists the path to the raw next-generation sequencing (FASTQ) files and index files from the generate_index step, as well as experimental information to produce processed files that can be used for several downstream steps (shown in yellow boxes).

We developed the Transcriptome Analysis Pipeline (TAP) to pre-processes raw sequencing data (fastq) across any reference genome (Fig. 1). The TAP pipeline begins with the build_index step that generates reference index files from user-supplied genome reference sequences and their corresponding annotation files (fasta and gtf, respectively). The build index process supports a variety of different annotation file formats, including NCBI, ENSEMBL and UCSC formats. Next, the user generates a configuration containing file and sample names, directory paths to the sequence and index files, and run parameters. The main pipeline can be executed to generate alignment, quantification, and signal files. TAP produces both gene and *Salmon* transcript- and *STAR* gene-quantification files that are useful for downstream differential expression analysis [19, 20], differential splicing analysis, and the detection of local alternative splicing patterns generated by *SUPPA2* [21]. Additionally, TAP produces stranded (if the library protocol is a stranded protocol) and read-depth normalized bigwig files to permit interactive visualizations of read pileup with a shareable genome browser session (i.e., UCSC genome browser) [22]. Finally, TAP also produces variant call files (VCF) containing single putative nucleotide polymorphisms (SNPs) and indels, which are produced as a part of the exactSNP tool from the *Rsubread* package [23]. These output files can be used for comprehensive technical and functional assessment of RNA-seq data, including quality control, differential expression, and alternative splicing. We designed the TAP pipeline to run on a high-performance computing cluster or parallel processing in the UNIX environment, utilizing Docker container technology and popular bioinformatics open-source software. TAP is freely available at https://github.com/FrietzeLabUVM/TAP.

### A comparison of library protocols for bulk RNA-seq analysis using whole D. melanogaster

**Figure 2:**
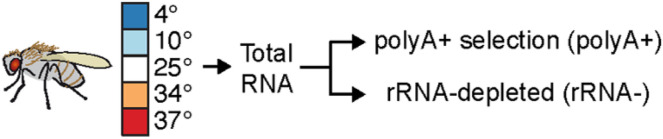
Experimental design to compare RNA-seq library types for studies involving *Drosophila melanogaster*. Adult female adult flies (9) were subject to different temperatures for 5 minutes and frozen for RNA extraction and construction of polyA+ selected and rRNA-depletion strand-specific RNA-seq libraries.

To evaluate RNA-seq data from different library protocols, we generated polyA+-selected (polyA+) and rRNA-depleted (rRNA−) libraries from *D. melanogaster* samples from adult female flies. These flies were subject to 5-minute incubation periods at temperatures ranging from 10° to 37°C, with three independent biological replicates (Fig. 2). These temperatures were chosen to induce both cold shock (4° and 10°C) and heat shock (34° and 37°C). We used the Transcriptomic Analysis Pipeline (TAP) to process RNA-seq data and assess quality control and functional transcriptomic measures. Reads were processed against the *D. melanogaster* DM6 reference transcriptome and the current FlyBase annotation (v5.23) was used to calculate transcripts per million (TPM) for all 35,733 annotated DM6 transcripts.

**Figure 3:**
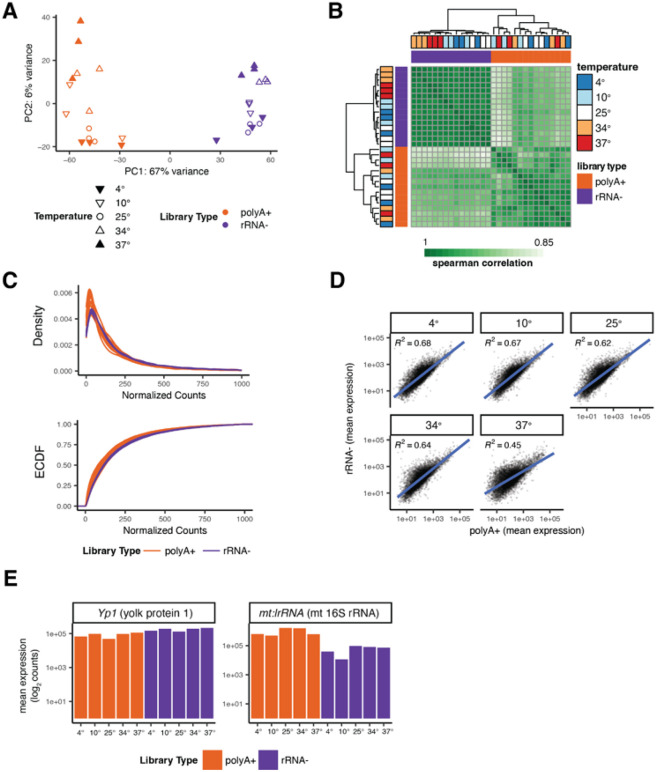
Quality control analysis comparing RNA-seq library protocols across temperature treatment conditions. **A)** Principal component analysis showing principal component 1 (PC1) and PC2 for RNA-seq data from flies treated at the indicated temperatures. **B)** Correlation heatmap of normalized RNA-seq data. Spearman correlation analysis and hierarchical cluster illustrates grouping patterns. **C)** Density plot of normalized expression count values of RNA-seq data (left) and empirical cumulative distribution function (ECDF) values for both library types. **D)** Pairwise scatterplot comparing the mean normalized expression values of expressed genes for rRNA-depleted and poly A+-selected libraries for each condition. Shown are the R^2^ values for each comparison.

Sample correlation was assessed by principal component analysis (PCA) and Spearman rank correlation analysis (Fig. 3A and 3B). These results showed that the RNA-seq datasets grouped together by library type and replicates from temperature groups clustered together. Both polyA + and rRNA− libraries showed overall similar read count densities across samples from different temperature and library conditions (Fig. 3C). Sample pairs for expressed transcripts from each temperature group between different library types were overall well correlated (Fig. 3D). The gene encoding Yolk protein 1 (*Yp1*, aka vitellogenin) was the most abundantly expressed transcript in rRNA− samples, while the mitochondrially encoded large ribosomal subunit rRNA (16S rRNA) was the most abundantly expressed transcript in polyA + samples (Fig. 3E).

**Figure 4:**
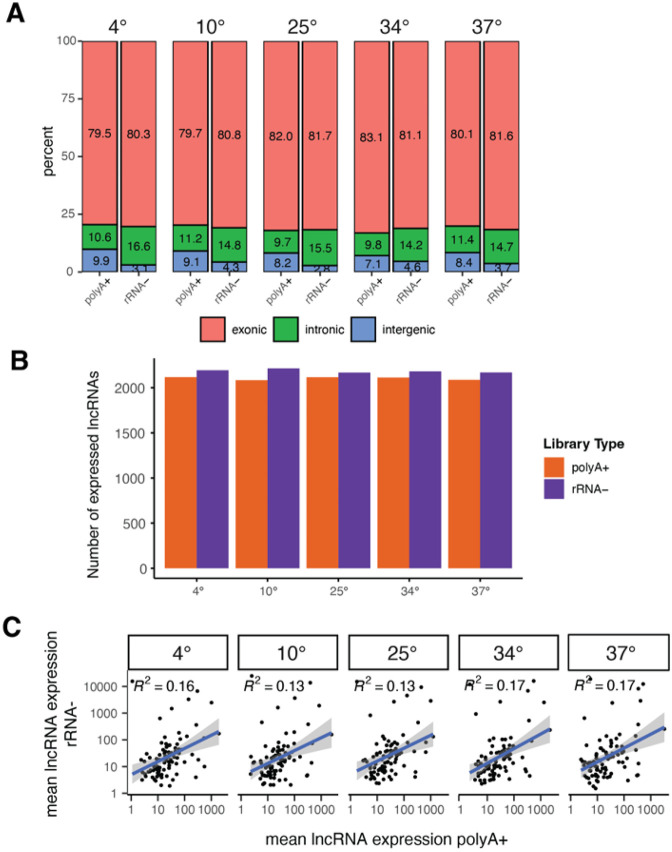
Comparison of mRNA and lncRNA expression between library types. **A)** Plot showing the fraction of total reads that map to exonic, intronic and intergenic gene features for both library types across the different temperature groups. **B)** Plot showing the number of annotated lncRNAs (TPM > 2) expressed in each sample. **C)** Pairwise scatterplot of mean normalized lncRNA expression between different library types.

We next evaluated the mapping of reads to various genomic regions features including exonic, intronic and intergenic regions in the different datasets. Reads that map to exonic regions were the most common in both library types, whereas rRNA− libraries had higher levels of intronic transcripts than polyA + libraries. In contrast, polyA + libraries showed higher levels of intergenic transcripts (Fig. 4A). We next evaluated the expression of long non-coding RNAs (lncRNAs) in the different library types and compared the expression levels of the 2,496 annotated lncRNAs from Flybase across library types. There were overall a similar number of expressed lncRNAs (mean TPM > 2) detected in polyA + and rRNA− across each temperature group, where approximately 80% of annotated lncRNAs were detected in each library type (Fig. 4B). However, the correlation of lncRNA expression levels at each temperature showed overall poor correlation between polyA + and rRNA− library types (Fig. 4C).

### Effect of library type on differential gene expression

**Figure 5:**
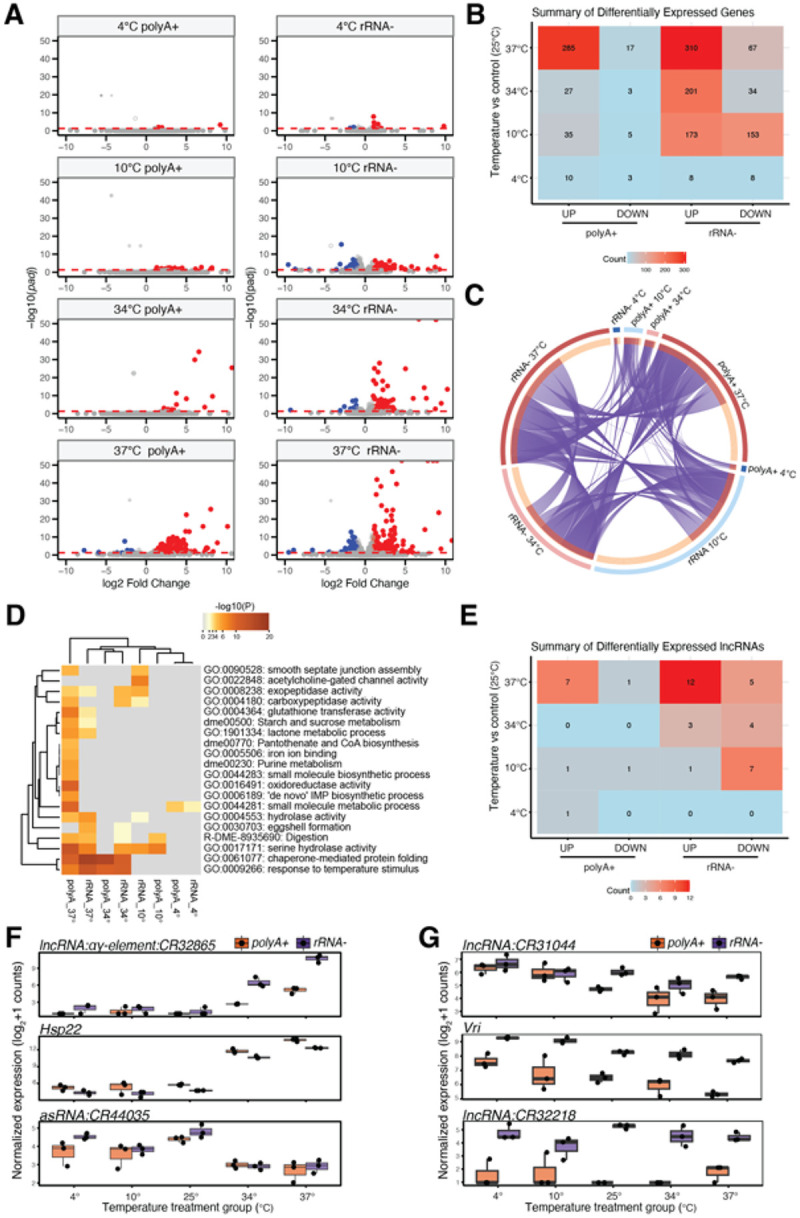
Comparison of differential gene expression patterns between library types. **A)** Volcano plots showing differentially expressed genes (DEGs) for each temperature group compared to control (25°C) conditions for each library type. Significantly up- and down-regulated DEGs (padj < 0.05 & fold-change > 2) are colored in red and blue, respectively. **B)** A heatmap summary of all up- and down-regulated DEGs are shown. **C)** The gene-level overlap among the DEG lists is represented by a Circos plot, where the color corresponds to different temperature treatment groups and library types. **D)** A clustered heatmap showing the results from pathway enrichment analysis performed on the indicated DEG groups. The color scale indicates the significance of enrichment by adjusted p-value. **E)** A heatmap summary of all up- and down-regulated differentially expressed lncRNAs (padj < 0.05 & fold-change > 2) are shown. **F) and G)** Expression plots showing differentially expressed genes identified from different comparisons (see results text). Shown are plots of the normalized expression counts (log_2_+1) for each group, colored by library type (polyA+ in orange and rRNA− in purple, respectively).

Differential gene expression analysis was next performed to compare the gene expression changes between cold or hot treatment groups and control (25°C), comparing polyA + and rRNA− libraries. Differentially expressed genes (DEGs) were identified in all comparisons, revealing distinct patterns of gene expression alterations under varying thermal stress conditions (Fig. 5A). We determined the number of DEGs for different temperature conditions and library types to expose varying degrees of gene regulation in response to thermal stress. As expected, the 37°C treatments exhibited the highest number of differentially expressed genes (DEGs) for both polyA + and rRNA− libraries. Notably, the 37°C treatment groups showed a similar number of upregulated DEGs for both polyA + and rRNA− libraries (285 and 310, respectively), however there were relatively more downregulated DEGs observed with rRNA− libraries (67 versus 17) (Fig. 5B). Similarly, both the 10°C and 34°C treatment groups had a higher number of DEGs for rRNA− compared to the corresponding polyA + libraries. To compare the relationships among DEGs, we compared the overlap of DEGs across each condition. Overall, there was a high degree of concordance between protein coding DEGs for both types of libraries across temperature groups (Fig. 5C).

We further compared the differentially expressed genes by performing Gene Ontology (GO) enrichment analysis. The significantly enriched GO categories for polyA + or rRNA− library types were largely similar among treatment groups (Fig. 5D). However, different library types for cold and hot treatment groups also showed distinctions in enriched categories, whereby several GO categories were unique to different library types across different temperature treatments (**S. Table 2**). For example, the 34° and 37°C heat treatments were enriched in response to temperature categories including and chaperone-mediated protein folding and serine hydrolase categories, whereas the 37°C polyA + dataset had several additional categories not significantly enriched in the 37°C rRNA− datasets such as purine metabolism and small molecule metabolic process. The 10° and 34°C rRNA− also had a higher number of unique enriched categories such as exopeptidase and carboxypeptidases, than the corresponding polyA + datasets. This analysis highlights the biological processes and molecular functions that are significantly affected by thermal stress and library preparation methods.

We next compared the differential expression of lncRNAs across different conditions between library types. The rRNA− libraries resulted in a greater number of differentially expressed lncRNAs for each temperature group, with the highest number from 37°C samples (17 total differentially expressed lncRNAs) (Fig. 5E). Differentially expressed lncRNAs with heat included *lncRNA:ag-element:CR32865*, which is induced at 34° and 37°C conditions compared to control or cold treatments in data from library types (Fig. 5F). These patterns mirrored expression of heat shock genes such as *Hsp22*, also known to be induced with thermal stress [31,32]. The antisense transcript *asRNA:CR44035* was among the list of lncRNAs significantly downregulated with heat in both libraries. Among the differentially expressed lncRNAs with cold stress included *lncRNA:CR31044* that showed elevated expression at cold temperatures (4° and 10°C) for both library types (Fig. 5G). This pattern of cold induction mirrored the expression of cold-induced mRNAs such as vrille (*Vri*) encoding a bZIP transcriptional repressor and clock-controlled circadian rhythm gene previously reported as a cold induced gene [32,33]. However, the effect of library protocol was evident in the expression analysis of lncRNAs such as *lncRNA:CR32368*, which was downregulated at 10°C compared to the other temperatures only in rRNA− libraries. The normalized expression of this transcript was detectable only in rRNA− libraries and showed very low expression in polyA + libraries (Fig. 5H).

### Effect of library type on transcript isoforms and local alternative splicing events

**Figure 6:**
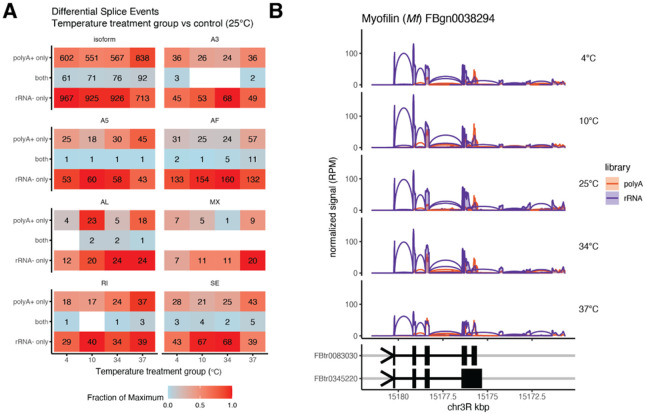
Splicing analysis comparing RNA-seq library protocols across temperature treatment conditions. **A)** A comparison of differential transcript isoforms and local alternative splicing events across temperature treatment groups and library types. Local splicing events included skipped exon (SE), alternative 5'/3' splice sites (A5/A3), mutually exclusive exons (MX), retained intron (RI), and alternative first/last exons (AF/AL). **B)** Analysis of splicing patterns based on normalized RNA-seq signal of the (−) strand (reads per million (RPM)) and transcript isoform annotations for the *Myofilin*gene (FBgn0038294) chr3R:15,170,625-15,181,510.

Alternative splicing is fundamental in the regulation of gene expression and can be systematically analyzed in RNA-seq data [34]. The TAP pipeline applies an integrative approach to enable analysis of transcript isoforms, differential alternative splicing events, and clusters of splice junctions between conditions. We compared transcript isoforms and local alternative splicing events between cold or hot treatment groups and control (25°C) for both polyA + and rRNA− libraries. We identified hundreds of differentially expressed transcript isoforms and local splicing events across different temperature conditions compared to controls for both library types (Fig. 6A). This is overall consistent with broad splicing changes known to occur in response to thermal stress [35]. Approximately, 10% of identified isoform changes were consistent in both library types in this data derived from whole animals. For example, we identified changes in transcript isoforms derived from the *Myofilin* (*My*; FBgn0038294) gene encoding the muscle-specific thick-filament associated protein known to be regulated by alternative splicing [36] (Fig. 6B).

## Methods

### Study animals

Canton S flies were obtained from the Bloomington Drosophila Stock Center (Bloomington, IN, USA). Prior to temperature exposure, flies were maintained at 25°C at L:D 12:12 and fed a standard cornmeal and soy flour diet consisting of 0.84% agar, 7.79% yeast, 9.35% cornmeal, 0.31% Tegosept (w/v) (US Biological, Swampscott, MA, USA), 4.36% molasses, and 0.62% propionic acid (v/v) in H_2_O. Flies were maintained in vials at a density of 30–50 flies per vial.

### Temperature shock regime

Adult females were exposed to cold or heat shock conditions by placing 6 flies in sealed 15 × 150 mm glass test tubes and submerging in a circulating water bath. The bath was programmed to cool or heat at a rate of 0.25°C/min to one of the four following temperatures: 4°C (severe cold shock), 10°C (moderate cold shock), 34°C (moderate heat shock) or 37°C (severe heat shock). 10°C and 34°C samples were removed once the bath reached the corresponding temperature. Flies were held for 5 minutes at 4°C or 37°C before being removed. Flies were anesthetized with CO_2_ and transferred to microcentrifuge tubes filled with zirconium silicate beads. These tubes were immediately snap-frozen in liquid nitrogen and held at −80°C until RNA isolation. Control flies were similarly handled but remained at 25°C until collection and flash-freezing.

### RNA isolation

Whole flies were homogenized using a Bullet Blender Bead Homogenizer (Next Advance, Troy, NY, USA) in 300 μL TRIzol Reagent (Life Technologies, Carlsbad, CA, USA). RNA was extracted according to the manufacturer’s instructions. Total RNA was resuspended in DNAse/RNAse-free water and quantified with a Nanodrop 2000 spectrophotometer (ThermoFisher, Waltham, MA, USA). RNA quality was assessed on a Bioanalyzer 2100 (Agilent Technologies, Santa Clara, CA, USA).

### RNA-seq library construction and sequencing

Strand-specific RNA-seq libraries were constructed following the stranded polyA+ (Illumina TruSeq) or a stranded rRNA-depletion kit (Tecan, Switzerland). > 800 ng was used in the polyA + libraries and 300 ng was used for the rRNA-depleted libraries, according to manufacturer’s instructions. The quantity and quality of the libraries were assessed with the DNA High Sensitivity Kit (Agilent Technologies) on a Qubit Fluorometer (ThermoFisher) and a Bioanalyzer 2100 (Agilent Technologies). The libraries were pooled and sequenced on an Illumina NovaSeq 6000 (polyA+) or an Illumina HiSeq 2000 (rRNA-depletion) with 150-bp and 85-bp read length, respectively at a sequencing depth of > 5 million reads per library (S. Figure 1).

### Processing of RNA-seq

The quality of the raw sequence data was examined using FastQC software (version 0.12.0) and visualized with MultiQC [24, 25]. To compare both library types, read lengths were standardized by trimming the raw polyA + sequencing files using trimmomatic [26] and aligned to the *D. melanogaster* reference genome (DM6). Transcript-level abundances of annotated transcripts for each sample were derived from the DM6 reference transcriptome using Salmon (version 1.4.0) [20] and imported into R/Bioconductor with *tximport* [27]. *DESeq2* [28] was used to estimate mean and dispersion parameters for a Negative Binomial distribution for gene-level counts for samples, and to perform differential expression analysis. Significantly differentially expressed genes were defined as genes with those that exhibit greater or equal to fold-change of 2 or padj < 0.05.

### Pathway enrichment analysis

Metascape multi-gene lists corresponding to the identified DEGs were used for functional enrichment analysis with default settings [29].

### Splicing analysis

The Ensembl Berkeley Drosophila Genome Project (BDGP) assembly release 6 (July 2014) [30] and the gene annotation file BDGP (version 110) were downloaded in GTF format from the Ensembl FTP server. We applied SUPPA to each annotation to obtain events from Ensembl, including exon skipping (SE), alternative 5′ and 3′ splice sites (A5/A3), mutually exclusive exons (MX), and intron retention (RI), Alternative first (AF) and last exons (AL).

## Discussion

In this study, we examined mRNA and lncRNA expression under varying thermal stress conditions in *Drosophila melanogaster*. This investigation was motivated by the growing recognition of the diverse and pivotal roles played by lncRNAs in cellular stress responses [13, 14]. Previous research in *D. melanogaster* had already shed light on the impact of thermal stress on various physiological, biochemical, and cellular processes [15], as well as the expression of stress response genes [16-18]. However, understanding lncRNA expression as potential regulatory mechanisms governing stress responses has been hindered by differences in experimental conditions (such as stress intensity or duration), tissue sampling, and variations in library preparation.

We evaluated the performance of two RNA-seq library protocols, polyA + and rRNA−, in the context of detecting gene and transcript expression under varying thermal stress conditions. One key aspect of this study was assessing the quality of these library types. We found that both library types demonstrated similar sensitivity and dynamic range, indicating their ability to effectively capture a wide range of gene expression levels. This consistency in performance highlights the reliability of both library protocols for quantifying gene and transcript expression across the conditions studied. We further examined the correlation of lncRNA expression between polyA + and rRNA− libraries. The calculated R^2 values were found to be relatively high (> 0.58 across conditions), suggesting an overall agreement in the expression levels of lncRNAs between the two library types (Fig. 4). These findings provide confidence in the suitability of both polyA + and rRNA− library protocols for comparable sensitivity of lncRNA expression in *D. melanogaster*.

Despite overall similar detection of gene expression, our analysis also revealed significant distinctions between the polyA + and rRNA− library protocols. Notably, the efficiency of rRNA removal from the samples using the commercially available kit was suboptimal and each rRNA− library had a large fraction of total reads that mapped to rRNA genes. Thus, in our analysis we performed rigorous quality control analysis by removing rRNA reads and normalizing by both useable read depth and read lengths (see Methods). However, this had a cost in read depth and resulted in approximately 2 million reads per library (Supplemental Fig. 1). Nevertheless, comparing usable reads, showed that the rRNA− protocol exhibited a heightened sensitivity to intergenic regions and intronic regions, as evidenced by the increased detection of intronic transcripts, particularly under extreme temperature conditions. In contrast, the polyA + libraries exhibited higher degree of exonic reads. The latter was manifested in a more robust detection of splice junctions in polyA + libraries.

Further distinctions between library types were revealed by performing differential gene expression analysis. We observed consistent changes in the expression of mRNAs associated with stress responses, including enriched in metabolic processes aligning with previous reports [37]. However, the polyA + protocol exhibited many additional enriched categories including those enriched in distinctive pathways including purine metabolism and small molecule metabolic process (Fig. 4). A comparison of DEGs across conditions between each library type revealed pathways associated with stress responses, including enrichment cellular responses to temperature category (GO:0009266) and involving induction of *Hsp22* encoding a heat shcok chaperone protein (Fig. 5). These DEGs showed consistent gradient of expression between heat shock conditions (34 and 37°C). Notably, rRNA− libraries showed an increased number of differentially expressed lncRNAs, particularly under extreme temperature conditions. This observation prompts further exploration into the regulatory roles of these differentially expressed lncRNAs during thermal stress.

This analysis unveiled the modulation of previously uncharacterized genes in response to thermal stress, encompassing both cold and heat-induced lncRNAs. The rRNA-depletion libraries had the highest number from 37°C samples for a total of 17 differentially expressed lncRNAs. Heat-induced lncRNA *lncRNA:ag-element:CR32865* expression significantly increased at 34° and 37°C treatments in data from both library types. However, the effect of library protocol was evident in the expression analysis of certain transcripts. For example, *lncRNA:CR32368* was downregulated at cold temperatures but only in rRNA− libraries. These observation prompts further investigations into the regulatory roles of lncRNAs during thermal stress.

Overall, our study showcased the effectiveness of the polyA + protocol in capturing the majority of expressed lncRNAs in *D. melanogaster*, highlighting its suitability for this biological system. In contrast, the advantages of rRNA depletion appeared comparatively limited under these conditions, particularly without optimization of the rRNA depletion. Beyond the technical aspects of library protocol comparison, our study has illuminated the complexities of lncRNA expression under thermal stress conditions, a critical facet of biological regulation. The differential gene expression analysis revealed distinct responses to temperature fluctuations, with rRNA− libraries exhibiting heightened sensitivity to temperature-induced changes. This underscores the imperative need to select an appropriate library protocol tailored to the specific research question, biological system, and desired resolution. The findings presented here also underscore the value of integrative bioinformatics pipelines like TAP. As researchers continue to explore diverse organisms and experimental conditions, a standardized and adaptable pipeline such as TAP can guide the processing and analysis of transcriptomic data. To facilitate broader accessibility and reproducibility, we have made TAP available through Docker, ensuring that other researchers can readily adopt and apply this valuable tool.

## Conclusions

Our investigation into the impact of library protocols on lncRNA expression in *D. melanogaster* underscore the significance of selecting an appropriate library protocol, not only to achieve accurate quantification but also to capture the complexities of gene expression patterns under distinct experimental conditions.

As the field of genomics evolves, our study contributes valuable insights into the interpretation of RNA-seq data, emphasizing the importance of thorough evaluations and the relevance of adaptable analysis pipelines.
